# MR-guided focused ultrasound technique in functional neurosurgery: targeting accuracy

**DOI:** 10.1186/2050-5736-1-3

**Published:** 2013-04-25

**Authors:** David Moser, Eyal Zadicario, Gilat Schiff, Daniel Jeanmonod

**Affiliations:** 1 Center of Ultrasound Functional Neurosurgery, Leopoldstrasse 1, Solothurn, CH-4500, Switzerland; 2 InSightec Ltd, Tirat Carmel, 39120, Israel

**Keywords:** Anterior commissure, Posterior commissure, Stereotactic atlas, Targeting accuracy, Thalamo-ventricular border, Transcranial MR-guided focused ultrasound

## Abstract

**Background:**

The purpose of this study was to describe targeting accuracy in functional neurosurgery using incisionless transcranial magnetic resonance (MR)-guided focused ultrasound technology.

**Methods:**

MR examinations were performed before and 2 days after the ultrasound functional neurosurgical treatment to visualize the targets on T2-weighted images and determine their coordinates. Thirty consecutive targets were reconstructed: 18 were in the central lateral nucleus of the medial thalamus (central lateral thalamotomies against neurogenic pain), 1 in the centrum medianum thalamic nucleus (centrum medianum thalamotomy against essential tremor), 10 on the pallido-thalamic tract (pallido-thalamic tractotomies against Parkinson's disease), and 1 on the cerebello-thalamic tract (cerebello-thalamic tractotomy against essential tremor). We describe a method for reconstruction of the lesion coordinates on post-treatment MR images, which were compared with the desired atlas target coordinates. We also calculated the accuracy of the intra-operative target placement, thus allowing to determine the global, planning, and device accuracies. We also estimated the target lesion volume.

**Results:**

We found mean absolute global targeting accuracies of 0.44 mm for the medio-lateral dimension (standard deviation 0.35 mm), 0.38 mm for the antero-posterior dimension (standard deviation 0.33 mm), and 0.66 mm for the dorso-ventral dimension (standard deviation 0.37 mm). Out of the 90 measured coordinates, 83 (92.2%) were inside the millimeter domain. The mean three-dimensional (3D) global accuracy was 0.99 mm (standard deviation 0.39 mm). The mean target volumes, reconstructed from surface measurements on 3D T1 series, were 68.5 mm^3^ (standard deviation 39.7 mm^3^), and 68.9 mm^3^ (standard deviation 40 mm^3^) using an ellipsoidal approximation.

**Conclusion:**

This study demonstrates a high accuracy of the MR-guided focused ultrasound technique. This high accuracy is due not only to the device qualities but also to the possibility for the operator to perform on-going real-time monitoring of the lesioning process. A precise method for determination of targeting accuracy is an essential component and basic requirement of the functional neurosurgical activity, allowing an on-going control of the performed therapeutic work indispensable for any target efficiency analysis and the maintenance of a low risk profile.

## Background

The therapeutic application of any technology to functional neurosurgery requires refined target reconstructions and precise targeting accuracy measurements, which should be within the millimeter domain. As targets are in normal tissue, coordinates have to be established for each target on the basis of a stereotactic atlas of the human brain. Such an atlas uses internal landmarks to position a coordinate system onto the brain, allowing the placement of any desired target inside the brain. We use the Morel *Stereotactic Atlas of the Human Thalamus and Basal Ganglia*[1,2], which provides, better than other currently used atlases, the following essential qualities: (1) a special guillotine has been used to guarantee proper cutting angles, (2) stereotactic maps were drawn every 0.9 mm, (3) several histological staining techniques were used (multiarchitectonic atlas), and (4) the atlas is based on a histological experience from seven human autopsy brains, and the consecutive atlas maps were drawn from four hemispheres.

In functional neurosurgery, two steps of the treatment procedure need to be performed with precision inside the millimeter domain: (1) the projection, based on the atlas, of the three coordinates of a chosen target onto the intra-operative magnetic resonance (MR) imaging and (2) the application of heat in the chosen target. The determination of the three-dimensional position of this target on intra-operative images and of the therapeutic lesion on post-operative MR imaging allows then to establish the targeting accuracy of the whole therapeutic procedure.

The targeting accuracy measurement procedure presented here can be used in any functional neurosurgical setup. It has been developed in the context of a clinical study which has a goal to provide relief to patients suffering from chronic therapy-resistant functional brain disorders (neuropathic pain, Parkinson's disease, and essential tremor) by performing small therapeutic ablations in the thalamus and subthalamus using the ExAblate Neuro system (InSightec Ltd, Tirat Carmel, Israel). This device allows the incisionless transcranial MR-guided application of focused ultrasound energy (MRgFUS) into brain tissue. A first paper only on neurogenic pain patients [3] analyzed the targeting accuracy of this technique for 18 targets, and another paper [4] described our targeting experience and reconstruction method on the first 11 targets of the current project. In the following, we present our targeting accuracy measurements for the now available consecutive 30 focused ultrasound targets.

## Methods

The presented data are part of a study approved by the Ethics Committee of Kanton Aargau/Solothurn and Swissmedic (Study No. 2010/041).

### Atlas coordinate system

**Figure 1:**
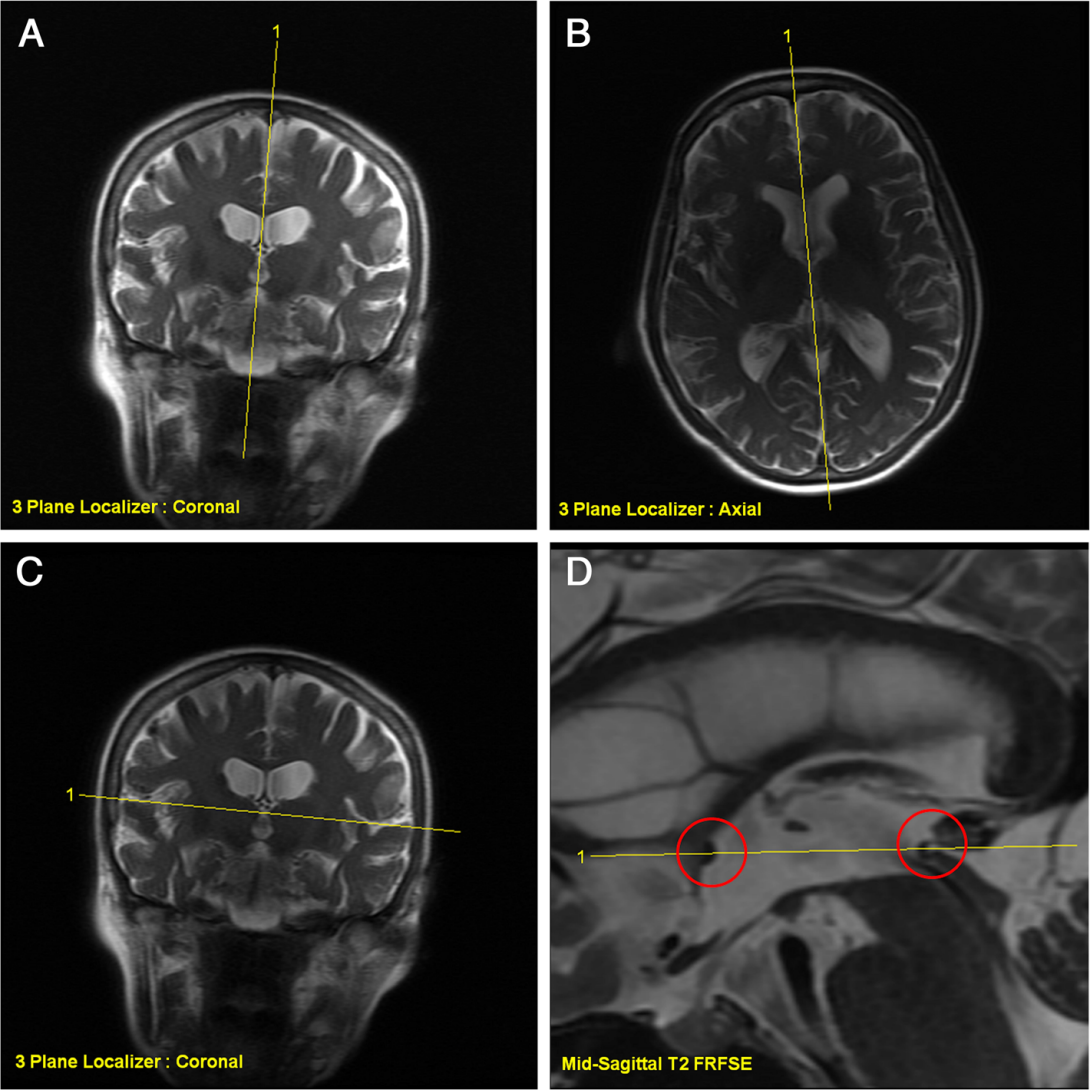
**MR prescription of the T2 FRFSE orthogonal sagittal and axial series.** Sagittal series is prescribed on three-plane localizer images, taking care of the tilting (**A**) and rotation (**B**) corrections for the planning of the mid-sagittal slice (D): the central prescription lines pass through the center of the third ventricle. Axial series is prescribed using three-plane localizer coronal (**C**) and the T2 FRFSE mid-sagittal (**D**) scans. The central prescription line on the mid-sagittal slice passes exactly through the center of the two commissures (*red circles* on D) and tilt is corrected on coronal slice (**C**).

**Figure 2:**
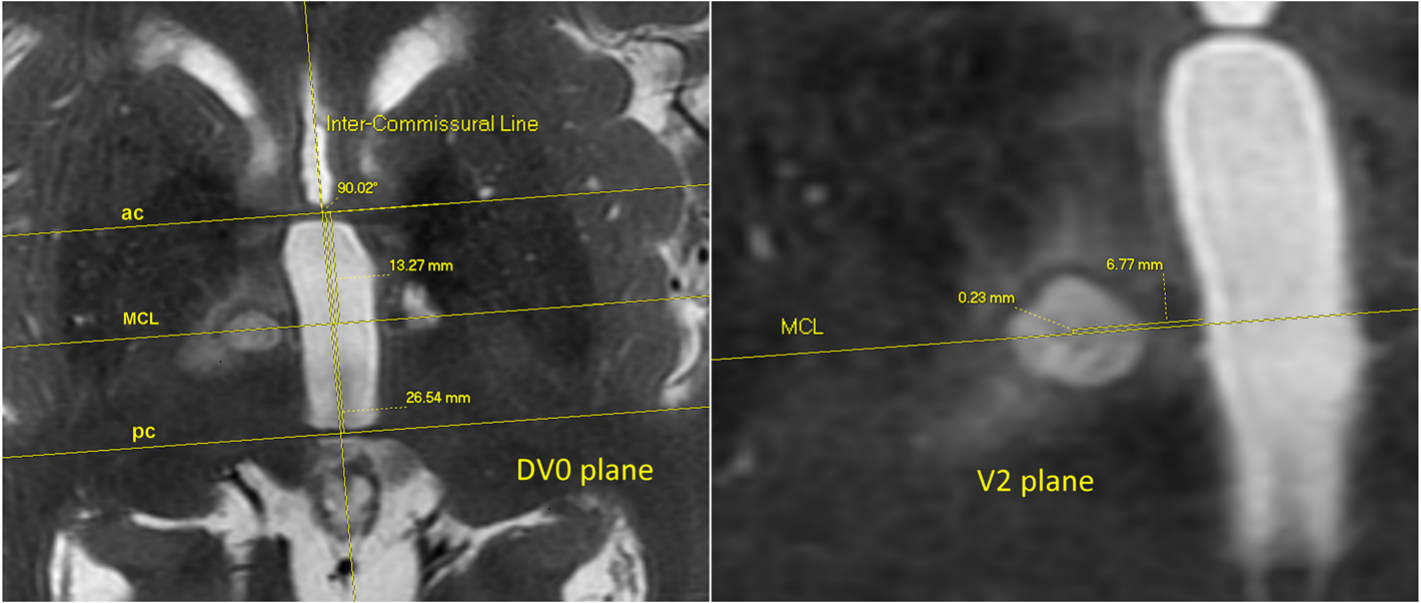
**Reconstruction of the realized target.** Realized target reconstruction (AP and ML coordinates of the center) on 2 days post-operative T2 FRFSE axial images. The reconstruction of the *ac* and *pc* lines and of the *MCL* is done on the DV0 plane (*left*). The ML coordinate is measured parallel to the MCL, while the AP direction is measured perpendicularly to this line on the V2 plane (*right*) where the target is most visible. The coordinates of the displayed realized target are 2 mm ventral to the ICL, 6.77 mm lateral to the thalamo-ventricular border, and 0.23 mm anterior to the MCL.

The neuroanatomical landmarks used in the Morel atlas [1,2] are the *anterior commissure* (ac) and the *posterior commissure* (pc). On a mid-sagittal scan (Figure 1D), a line passing through the center of these commissures (inter-commissural line, ICL) is used to determine the axial dorso-ventral (DV) ‘0’ plane. On an axial scan (Figure 2, left), the ICL is drawn passing through the middle of the third ventricle. The medio-lateral (ML) axes (right and left) are parallel to the ac and pc lines, themselves perpendicular to the ICL. On the atlas maps, the reference grid starts laterally in ‘0’ position in the middle of the ventricle. However, the width of the ventricle can vary significantly from patient to patient; the thalamo-ventricular border has thus been chosen as the reference point for the determination of the ML target coordinate (Figure 2, right).

### Targets and accuracies

There are three different kinds of targets, defined by the three-dimensional coordinates of their center, for each accuracy measurement: the chosen target, based on surgical experience and established in terms of atlas coordinates, has been called the *atlas target*. Following the atlas-based manual determination of the position of the target on the intra-operative MR images, its MR coordinates (in the right-anterior-superior (RAS) domain) are entered in the ExAblate software. These MR coordinates define the *prescribed target.* Finally, the position of the center of the therapeutic thermolesion, defined as the *realized target*, is measured on the MR post-operative exam.

On this basis, three accuracies can be computed: (1) the *global accuracy*, which is defined as the difference between the realized and the atlas target center coordinates, (2) the *device accuracy*, defined by the difference between the prescribed and the realized target center coordinates, and (3) the *planning accuracy*, defined by the difference between the atlas and the prescribed target center coordinates. It is to be noted that the device accuracy contains in fact (1) a purely *technical accuracy*, which depends on the construction of the machine and (2) a *manual correction* of the electronic steering (with its own accuracy) which can be applied by the operator during the treatment if the aiming of the machine is not on spot. Thus, the device accuracy may be considered as machine- as well as man-based. However, this man-based manual correction depends on the quality of the MR thermal maps and is therefore technically related.

Summarizing, the three accuracies that have been analyzed are the global, the device and the planning accuracies, which are linked as follows:

$$\begin{array}{cc} \;\mathrm{Global}\;\mathrm{accuracy} & =\mathrm{Device}\;\mathrm{accuracy}\\ & \;+\mathrm{Planning}\;\mathrm{accuracy} \end{array}$$

Each of these three accuracies is a three-dimensional signed vector and is calculated in the three dimensions: ML, antero-posterior (AP), and DV. The Euclidean length of the global accuracy vector is called the *3D global accuracy*.

When computing the mean over a group of patients, the ML, AP, and DV coordinates of their global accuracies should not compensate each other. For example, if a +1-mm difference on a direction is measured for one targeting and a -1-mm difference for another targeting in the same direction, the mean will be 0. The mean should however be 1 mm, and this is obtained by using the absolute value of the global accuracies of all patients.

### Tools

A brief description of the tools used to determine and reconstruct targets is shown on Table 1. The intra-operative target determination is done on the MR workstation, the post-operative prescribed target determination on the ExAblate workstation, and postoperative target reconstructions on a usual desktop computer (not mentioned in the table).

**Table 1:** Tools for targeting accuracy measurement

	**Tool**		**Use**
Hardware	InSightec ExAblate 4000 Neuro		FUS operation
	GE Discovery MR750 3.0 T	Body coil	Intra-operative setup
		32-channel head coil	Post-operative setup
Software	InSightec ExAblate FUS Brain software		Operation and post-operative prescribed target center measurement
	GE viewer software (on MR console)		Intra-operative target determination
	Carestream PACS V11.0 (DICOM viewer)		Realized target measurement on post-operative MR images
	Synedra View Personal 3 (DICOM viewer)		Volume measurements
	The Mathworks Matlab R2009b		Data processing

Tools used for target determination (intra-operative) and target reconstructions (post-operative).

### Imaging

The post-operative MR imaging was performed 2 days after the MRgFUS treatment. We have chosen this moment based on our year-long experience with radiofrequency (RF) lesioning and on our earlier observations [3] with MRgFUS, indicating that the visualization of the therapeutic lesion on MR pictures is optimal (i.e., full-blown) at this time after the thermocoagulation. The histological process of thermocoagulation is reproducible but shows nevertheless some variation along time, so that we cannot exclude a certain amount of inhomogeneity of lesion development at the chosen point in time.

The precision of all target determinations and reconstructions rely on MR imaging. If the three orientations are not orthogonal and precisely prescribed, a very significant error in target determination and measurements may happen. All MR series are cut in the same way as the Morel atlas [1,2] maps: the ‘zero’ axial plane passes through the centers of the ac and pc (Figure 1D). A minimum of two out of the three orientations (axial, sagittal, and coronal) is required in order to prescribe a target or to perform a target reconstruction. In the following, only the sagittal and the axial series have been used.

The sagittal scans are prescribed on the ‘three-plane localizer’ images, using axial and coronal orientations (Figure 1A,B). Care must be taken to prescribe a precisely mid-sagittal slice, thus allowing the detailed visualization of the ac and pc. This is performed mainly on a transthalamic three-plane localizer axial image: the prescription of the mid-sagittal slice must pass in the middle of the third ventricle with the proper angle (Figure 1B). The tilt of this plane has to be checked on the coronal three-plane localizer image (Figure 1A), adjusting also here the angle to be strictly mid-sagittal. The prescription of the axial series is then done using this mid-sagittal image and the same coronal three-plane localizer image. The central axial slice has to pass through the centers of the ac and pc (Figure 1D), while its tilt has to follow the patient's head tilt as seen on the coronal images (Figure 1C).

### Intra-operative atlas target determination

The atlas target coordinates are projected onto intra-operative transthalamic MR T2 fast relaxation fast spin echo (FRFSE) sagittal and axial series, with 2-mm thickness and a 0-mm gap between slices. 

Using the GE viewer software, the DV0 plane (slice containing the two commissures) is located on the axial series. On this slice, the ICL is drawn, passing in the middle of the third ventricle. Then, using the ‘angle tool,’ the ac and pc lines are drawn perpendicularly to the ICL and passing through the center of each commissure. The mid-commissural line (MCL) is drawn on this plane, at equal distance from the ac and pc lines (Figure 2, left). The AP target coordinate is positioned in relation to the one of the three landmarks - ac, pc, or mid-commissural point - which is closest to the target, with the goal to reduce the effect of the inter-individual variability (Figure 2, right). These lines are then copied on all the slices of the axial series.

The next step is to browse the axial series in order to find the right DV plane which depends on the DV coordinate of the atlas target. The AP position of the target is determined by drawing a line passing through the given AP coordinate, a line which is parallel to the ac, pc, and MCL*.* This line is then used to determine the ML position of the target, which is given starting from the thalamo-ventricular border. The target is now fully determined; the last point is to write down the RAS coordinates of the target in order to enter them into the ExAblate software.

### Realized target reconstruction

The position of the center of the lesion, or realized target, is determined on post-operative MR T2 FRFSE sagittal and axial series in essentially the same manner as for the intra-operative target determination. The post-operative MR series are the same as the ones used for the intra-operative target determination, except that a 32-channel head coil is now used instead of the MR body coil.

As explained above, the ICL and then the ac, pc, and MCL are drawn and copied on all the slices of the axial series. Then, the axial slice where the lesion is the most visible is located and the center of the lesion determined. On this particular slice, measuring the distance, parallel to the ac line, pc line, or MCL, from the center of the lesion to the thalamo-ventricular border gives the ML coordinate of the realized target. Measuring the distance between this center and the ac line, pc line, or MCL perpendicularly to them gives its AP coordinate.

The exact DV coordinate of the realized target is then determined using the sagittal series. On the mid-sagittal slice, the ICL is drawn and copied on all slices of the series. The slice where the lesion is most visible is located and the center of the lesion determined. The distance between this center and the ICL, perpendicularly to it, gives then the DV coordinate of the realized target.

### Prescribed target reconstruction

To check the accuracy of the placement of the target into the ExAblate software (RAS coordinates of the prescribed target), the ‘Replay Mode’ of the ExAblate workstation is used. The process is nearly the same as for the reconstruction of the center of the realized target; the only difference is that the blue rectangle representing the prescribed target has to be located instead of the lesion. The second, less important difference is that the Replay Mode of the ExAblate software is used instead of the GE viewer software on the MR console.

### Estimated lesion volume

**Figure 3:**
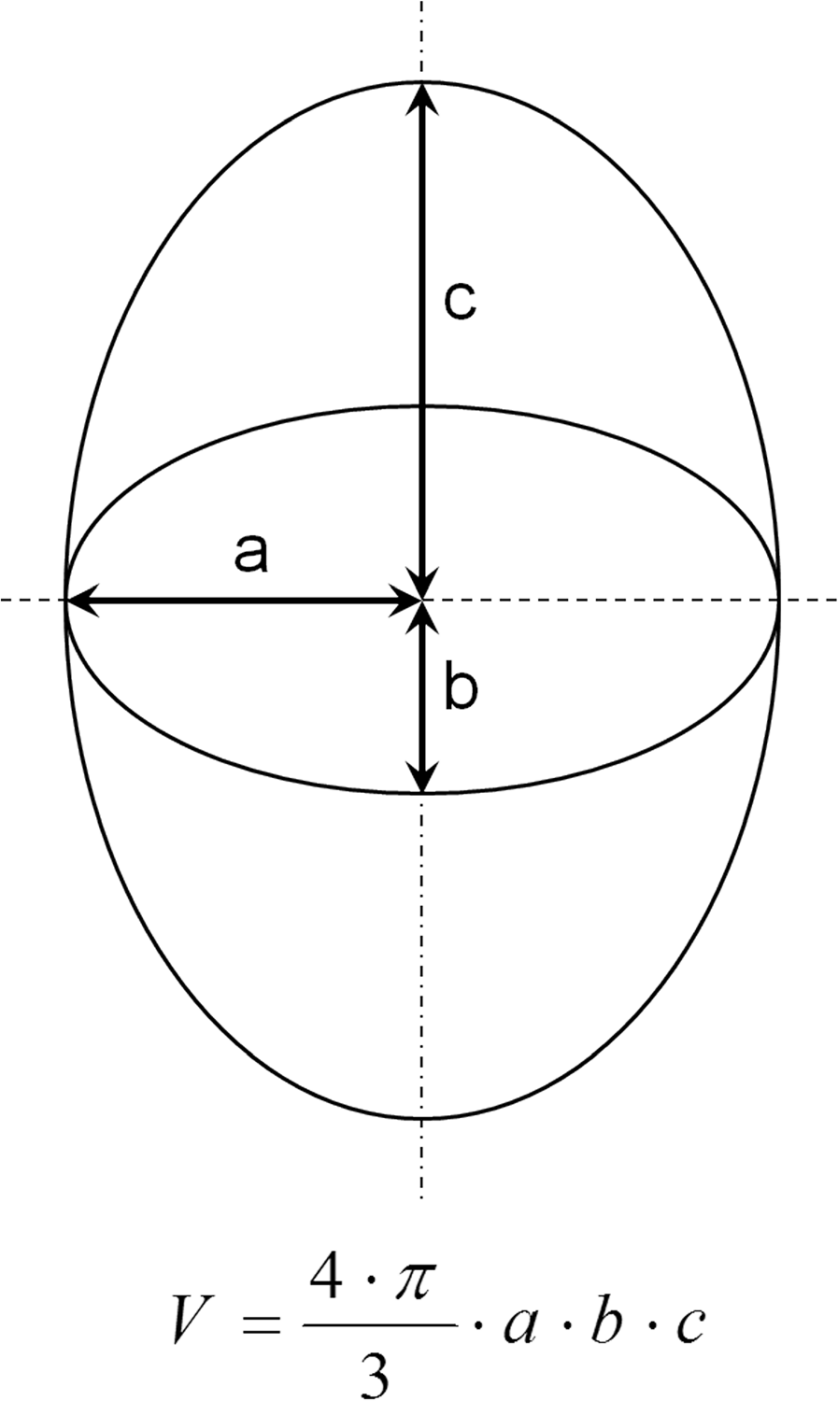
**Three-dimensional ellipsoid and its volume calculation.***Top*: three-dimensional ellipsoid with its half-axes *a*, *b*, and *c*. *Bottom*: equation of the volume of a three-dimensional ellipsoid.

The volume of the lesion could be estimated by measuring its diameter and height and computing the volume as if the lesion was a cylinder. The technique used is more accurate, as it compares the shape of the lesion to a three-dimensional ellipsoid (Figure 3). The first two half-axes (‘a’ and ‘b’ on Figure 3) are obtained by measuring the lesion diameters (and taking the half of it) on the axial scan where the lesion is the most visible. The last half-axis (‘c’ on Figure 3) is obtained by measuring the height of the lesion (and also taking the half of it) on a sagittal scan where it is the largest. In order to check the accuracy of the ellipsoidal approximation, the lesion volume has also been reconstructed from axial 3D T1 series using surface measurements of the lesion from all slices where it is visible.

## Results

**Figure 4:**
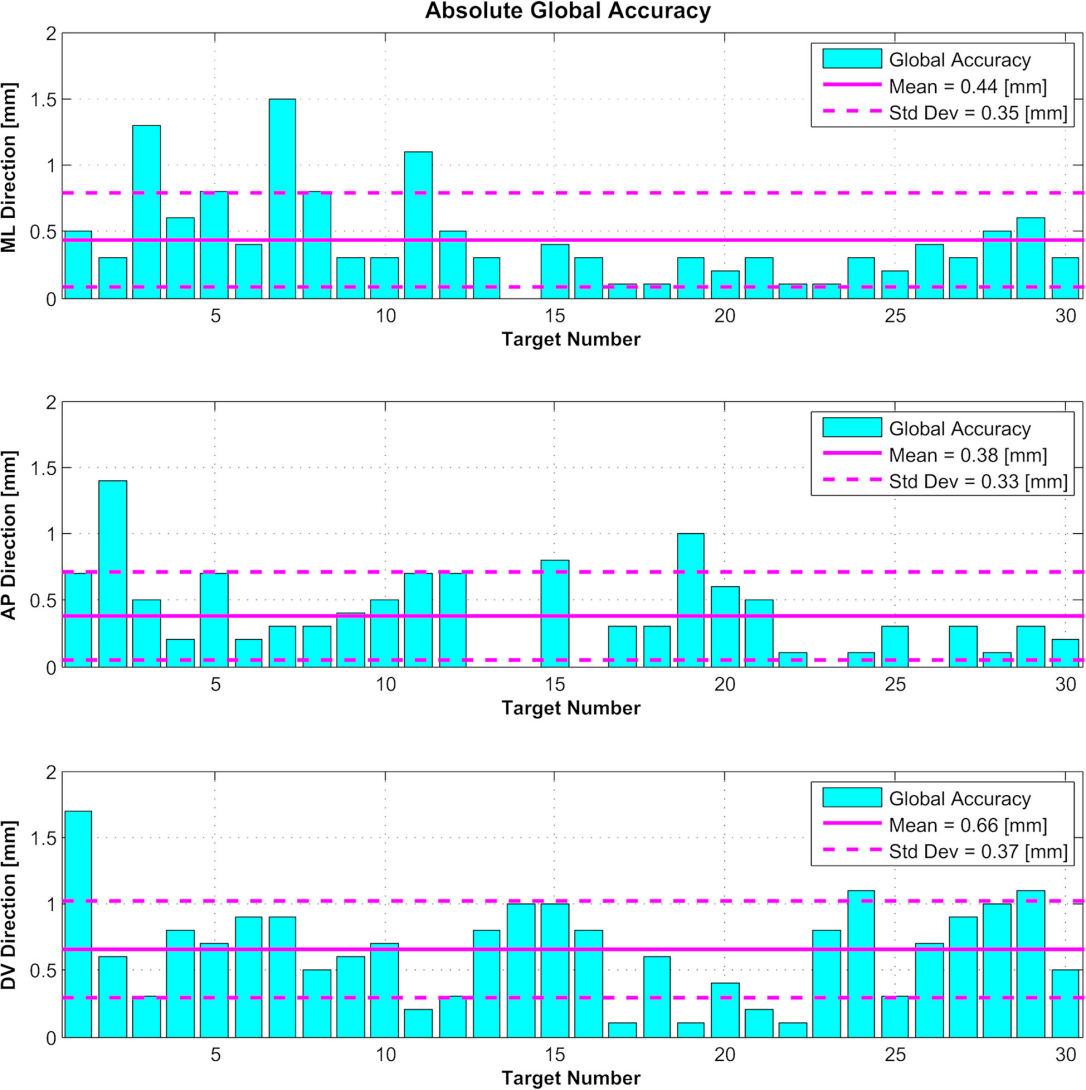
**Global targeting accuracy.** Absolute value of the global accuracies of the 30 reconstructed targets in the three directions, with the mean and standard deviation (*Std Dev*) for each. *ML* stands for medio-lateral, *AP* for antero-posterior, and *DV* for dorso-ventral.

**Figure 5:**
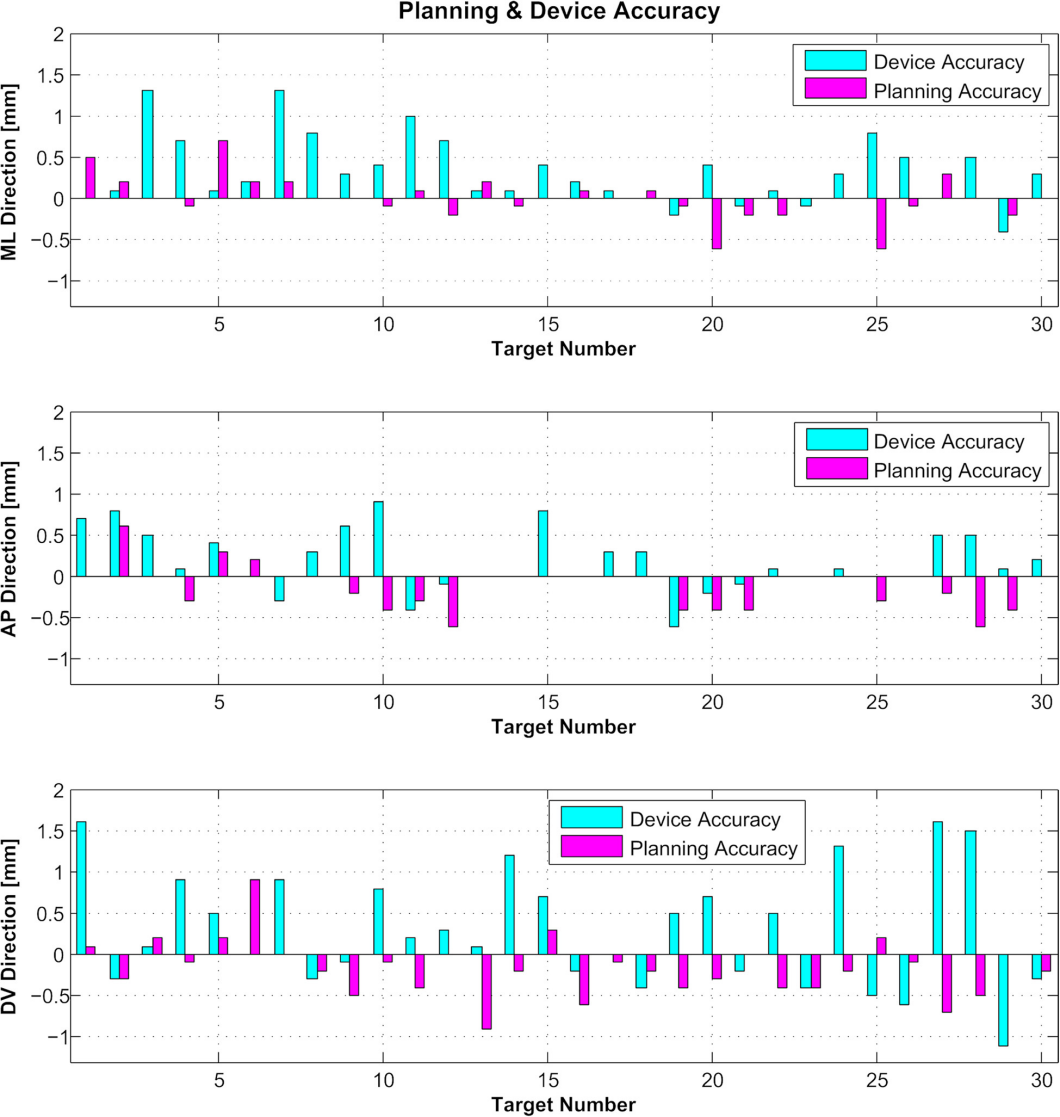
**Device and planning accuracies.** Device and planning accuracies for the 30 reconstructed targets. The sum of the absolute values of these two accuracies gives the global one. *ML* stands for medio-lateral, *AP* for antero-posterior, and *DV* for dorso-ventral.

**Figure 6:**
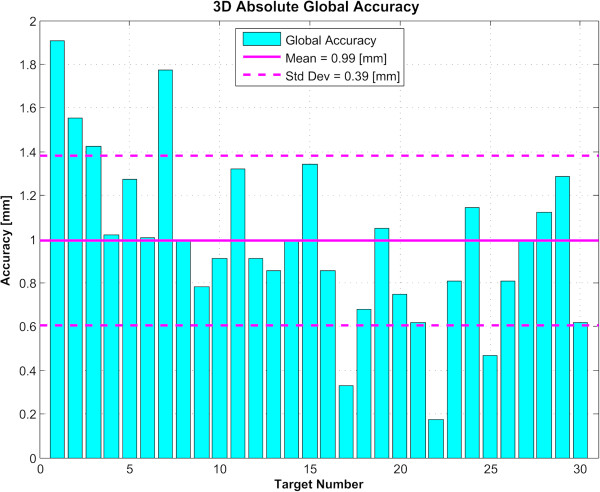
**Three-dimensional global targeting accuracy.** Absolute value of the global *accuracies* of the 30 reconstructed targets in a three-dimensional space, with the mean and standard deviation (*Std Dev*).

For the 30 examined targets, we determined first the absolute global accuracy for each direction (Figure 4). The mean was 0.44 mm for the ML dimension (standard deviation 0.35 mm), 0.38 mm for the AP dimension (standard deviation 0.33 mm), and 0.66 mm for the DV dimension (standard deviation 0.37 mm). Over 90 measured coordinates, 7 (7.8%) exceeded the millimeter domain (maximum 1.6 mm). Figure 5 displays the planning and device accuracies of the 30 targets. The mean three-dimensional global accuracy (Figure 6) was 0.99 mm (standard deviation 0.39 mm).

**Figure 7:**
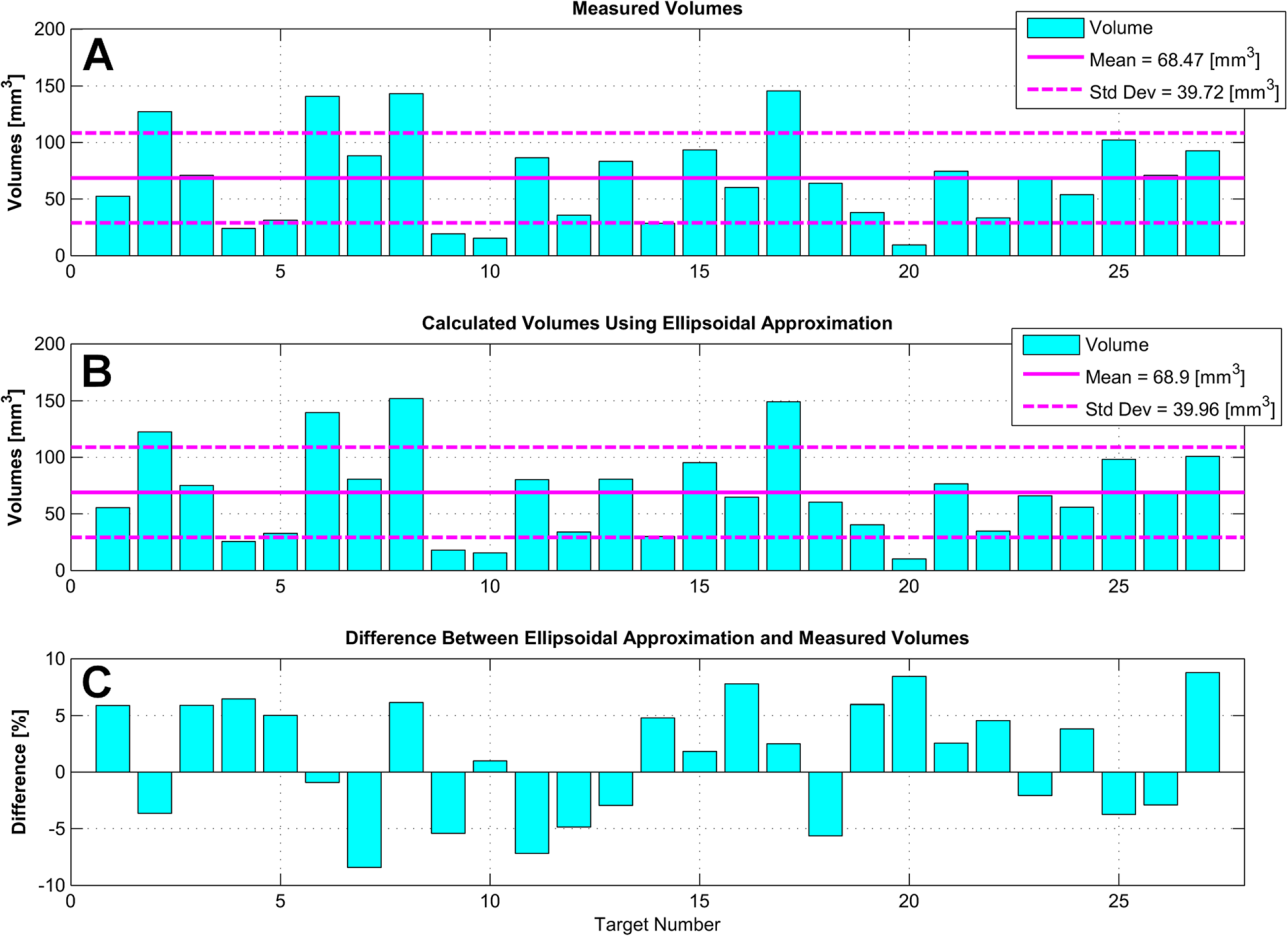
**Lesion volumes.** Volumes of the first 27 lesions with mean and standard deviation (*Std Dev*) using two different methods. (**A**) Volumes reconstructed from surface measurements on 3D T1-weighted post-operative scans. (**B**) Calculated volumes with the three-dimensional ellipsoid approximation. (**C**) Differences between the ellipsoidal approximations and the reconstructed volumes in percent.

Reconstructing the lesion volume from an axial 3D T1-weighted series, we obtained a mean lesion volume for the 27 first targets of 68.5 mm^3^ (standard deviation 39.7 mm^3^, see Figure 7A). Using the ellipsoid approximation, the mean volume of the first 27 lesions was 68.9 mm^3^ (standard deviation 40 mm^3^, see Figure 7B). The plot of the differences between the two techniques is shown in Figure 7C. The last three lesions have been dismissed from Figure 7 because they are larger due to target heating repetition (see the ‘Discussion’ section).

There were no device- or procedure-related complications and no post-treatment neurological deficits.

## Discussion

Along the last 23 years, our group has developed and applied as a routine target accuracy controls based on the Morel atlas [1,2]. This was warranted by our choice to perform selective small ablations based on detailed pathophysiological and neuroanatomical evidence [2,5-21].

We would like to stress here the importance of two factors contributing to adequate target determination and targeting accuracy measurement: first the proper alignment of the used MR slice stacks with correction of tilts and rotations, particularly relevant for lateral targets, and second the necessity of a high-resolution visualization of the two commissures, allowing a refined determination of their centers under high magnification. In our experience, the described T2-weighted imaging without 3D reformatting has been the best option, for post-operative imaging using a 32-channel head coil, but also for intra-operative imaging using the body coil of the GE Discovery MR750 system.

We have demonstrated here, using the described method for 30 targets, that 92.2% of our target coordinates lie within the millimeter domain, with a mean absolute global targeting accuracy of the focused ultrasound treatment between 0.38 and 0.66 mm and a mean 3D global accuracy of 0.99 mm. The MR-guided focused ultrasound technique fulfills thus the basic criterion of a mean accuracy smaller than 1 mm for the three coordinates, confirming two earlier studies [3,4] on 18 and 11 targets, respectively. It compares indeed favorably with accuracy studies of the deep brain stimulation (DBS) and RF techniques. In DBS, Bjartmarz and Rehncrona [22] found an error 3D vector of 2.5 mm (as compared to our value of 0.99 mm), and Hamid et al. [23] published errors of 0.48, 0.69, and 2.9 mm for the three axes (as compared to our values of 0.44, 0.38, and 0.66 mm). RF lesioning accuracy values were 0.8, 0.9, and 1.9 mm for the three axes in the study of Bourgeois et al [24]. These authors proposed that their DV accuracy of 1.9 mm is due to the mechanical shift of brain tissue, an interpretation fitting with the smaller value found in this study (0.66 mm), thanks to the absence of mechanical brain penetration. As for gamma knife, Massager et al. [25] published a 3-D vector value of 0.91 mm, close to our value of 0.99 mm.

The analysis of the lesion volumes applying an ellipsoidal approximation on T2-weighted imaging came out to be very close to lesion volume reconstructions from T1-weighted imaging (Figure 7). The ellipsoidal approximation may thus be proposed as a simple yet accurate estimation of the lesion volume. The three last measurements showed larger volumes because a repetition of the end temperature came out to be warranted to obtain a complete lesioning in fiber tracts, a measure which is not necessary in a nuclear (thalamic) area (Magara A. et al., unpublished work). These last three measurements have therefore been dismissed from the lesion volume analysis.

A precise method for determination of targeting accuracy is an essential component and basic requirement of the functional neurosurgical activity, allowing an on-going control of the performed therapeutic work indispensable for any target efficiency analysis and for the maintenance of a low risk profile. The applied target reconstruction procedure entails of course a certain amount of measuring errors, which we estimate on the basis of our experience to be each around half a millimeter. These errors are mainly variations of thalamo-ventricular border position due to differences of MR picture windowing, thickness of the MR slices (2 mm), and determination of the centers of the ac and pc and of the center of more or less regular ellipsoidal lesions. Most of these errors are related to the fact that the performed steps are based on individual visual and manual estimation. One limitation of our study is that we have not made an inter-individual estimation of measurement variations, another is that we have not used the coronal MR planes to countercheck our measurements on sagittal and axial series. In addition, variable amounts of perilesional edema may have influenced some measurements, particularly of the ML lesion coordinate.

The accuracy obtained with the MRgFUS system obeys clinical efficiency and safety criteria and is related to the device qualities but also to the possibility for the operator to manually correct the targeting in the context of an on-going real-time monitoring of the lesioning process. Future technological developments in the MRgFUS domain can be expected to contribute to further progresses in targeting precision, together with increased operator experience, considering the particular importance of human decision-making for an optimized treatment process.

## Competing interests

The current study is supported partially by InSightec Ltd (Haifa, Israel), Rodiag Diagnostics Centers AG (Olten, Switzerland), and GE Medical Systems (Switzerland). Eyal Zadicario and Gilat Schiff are employees of InSightec Ltd, manufacturer of the *ExAblate Neuro* System tested in the study.

## Authors' contributions

DM has contributed essentially to the conception and design of the study, carried out the acquisition, analysis, and interpretation of the data, and drafted the manuscript. EZ and GS contributed to the conception and design of the study and revised critically the manuscript. DJ contributed to the conception and design of the study and helped in the acquisition and interpretation of the data and in drafting the manuscript. All authors read and approved the final manuscript.
