# Correction: MR-guided focused ultrasound technique in functional neurosurgery: targeting accuracy

**DOI:** 10.1186/2050-5736-1-17

**Published:** 2013-09-27

**Authors:** David Moser, Eyal Zadicario, Gilat Schiff, Daniel Jeanmonod

**Affiliations:** 1 Center of Ultrasound Functional Neurosurgery, Leopoldstrasse 1, Solothurn CH-4500, Switzerland; 2 InSightec Ltd, Tirat Carmel 39120, Israel

## Correction

The authors would like to highlight the overlap in the Background, Methods and Discussions sections between their article in the *Journal of Therapeutic Ultrasound*[1] and their previous publication in *Neurosurgical Focus*[2], published and copyrighted by the American Association of Neurosurgeons. The novel aspects of this article [1] are the demonstration of the applicability and usefulness of the target reconstruction method described in [2], a quantification of target volumes using two novel approaches, and the publication, in the developing therapeutic focused ultrasound international environment, of the largest (30 targets) targeting accuracy analysis of MR-guided focused technique in functional neurosurgery. Table 1 in the article in [1] has been adapted from Table 1 in the *Neurosurgical Focus* article [2]. Figure 1 in [1] is also a composite of Figures 2 and 3 in [2]. We apologise to both Publishers, Editors-in-Chiefs of the journals and readers.

## References

[B1] Moser D Zadicario D Schiff G Jeanmonod D MR-guided focused ultrasound technique in functional neurosurgery: targeting accuracy J Ther Ultrasound 2013 1 3 10.1186/2050-5736-1-3 24761224 PMC3988613

[B2] Moser D Zadicario D Schiff G Jeanmonod D Measurement of targeting accuracy in focused ultrasound functional neurosurgery Neurosurgial Focus 2012 32 1 E3 10.3171/2011.10.FOCUS11252 22208895

